# Unveiling the Genomic Landscape of Intraductal Carcinoma of the Prostate Using Spatial Gene Expression Analysis

**DOI:** 10.3390/ijms25094818

**Published:** 2024-04-28

**Authors:** Ryuta Watanabe, Noriyoshi Miura, Mie Kurata, Riko Kitazawa, Tadahiko Kikugawa, Takashi Saika

**Affiliations:** 1 Department of Urology, Ehime University Graduate School of Medicine, Toon 791-0295, Japan; miura.noriyoshi.mk@ehime-u.ac.jp (N.M.); takikuga@m.ehime-u.ac.jp (T.K.); saika.takashi.ol@ehime-u.ac.jp (T.S.); 2 Human Biology Division, Fred Hutchinson Cancer Center, Seattle, WA 98109, USA; 3 Department of Analytical Pathology, Ehime University Graduate School of Medicine, Toon 791-0295, Japan; miekrt@m.ehime-u.ac.jp; 4 Division of Pathology, Proteo-Science Center, Ehime University, Toon 791-0295, Japan; 5 Division of Diagnostic Pathology, Ehime University Hospital, Toon 791-0295, Japan; riko@m.ehime-u.ac.jp

**Keywords:** spatial gene expression analysis, intraductal carcinoma of the prostate (IDCP), hypoxic markers, immune cells, tumor microenvironment

## Abstract

Intraductal carcinoma of the prostate (IDCP) has recently attracted increasing interest owing to its unfavorable prognoses. To effectively identify the IDCP-specific gene expression profile, we took a novel approach of characterizing a typical IDCP case using spatial gene expression analysis. A formalin-fixed, paraffin-embedded sample was subjected to Visium CytAssist Spatial Gene Expression analysis. IDCP within invasive prostate cancer sites was recognized as a distinct cluster separate from other invasive cancer clusters. Highly expressed genes defining the IDCP cluster, such as *MUC6*, *MYO16*, *NPY*, and *KLK12*, reflected the aggressive nature of high-grade prostate cancer. IDCP sites also showed increased hypoxia markers *HIF1A*, *BNIP3L*, *PDK1*, and *POGLUT1*; decreased fibroblast markers *COL1A2*, *DCN*, and *LUM*; and decreased immune cell markers *CCR5* and *FCGR3A*. Overall, these findings indicate that the hypoxic tumor microenvironment and reduced recruitment of fibroblasts and immune cells, which reflect morphological features of IDCP, may influence the aggressiveness of high-grade prostate cancer.

## 1. Introduction

Intraductal carcinoma of the prostate (IDCP) is a condition in which atypical cells from outside the glandular ducts invade and proliferate in normal glandular structures, leaving behind some basal cells and showing a cribriform morphology and extensive growth pattern. In 1996, McNeal et al. first reported IDCP with invasive cancer [1]. Since then, IDCP has received increasing attention because of its association with a poor prognosis. The presence of IDCP during radical prostatectomy has been reported to be associated with a higher Gleason score, larger tumor volume, extraprostatic extension, neuroendocrine differentiation, and accelerated disease progression [2,3,4,5]. Further, patients with localized and advanced prostate cancer (PCa) with IDCP have been reported to show significantly worse recurrence-free and overall survival rates [6,7,8,9].

For these reasons, IDCP was first described by the International Society of Urologic Pathology in 2014 [10] and subsequently included in the 2016 World Health Organization Prostate Tumor Classification [11], 2017 American Society of Pathology guidelines, and 2019 National Comprehensive Cancer Network (NCCN) guidelines [12].

Reports from Western countries have shown that homologous recombination (HR) gene mutations, including *BRCA* mutations, in addition to *TMPRSS2-ERG* fusion gene and *TP53*, *RB1*, and *PTEN* deletions, are frequently detected in patients with IDCP [13,14,15,16]. Interestingly, one study reported the presence of *BRCA2* mutations in approximately 42% of IDCP cases [17,18]; further, the NCCN guidelines state that early genetic testing should be considered in prostate cancer patients with IDCP. On the other hand, some reports deny the association between IDCP and HRR gene abnormalities, and a more adequate understanding of the pathogenesis of IDCP is needed [19]. Therefore, identifying IDCP-specific genetic abnormalities will contribute to the elucidation of molecular mechanisms underlying IDCP pathogenesis and the promotion of precision medicine.

Prostate cancer is extremely heterogeneous; therefore, gene expression analysis specific to IDCP lesions using conventional bulk gene analysis is impossible. Single-cell RNA-seq allows an understanding of tumor heterogeneity based on gene expression at the single-cell level. However, as IDCP is a morphology-based diagnosis, the application of single-cell RNA-seq is unsuitable because of the loss of spatial information. Therefore, in this study, we made a groundbreaking attempt to analyze the gene abnormalities characteristic of IDCP sites while maintaining the spatial information of IDCP using spatial gene expression analysis technology.

## 2. Results

### 2.1. Diagnosis of IDCP and Clustering Using Spatial Gene Expression Analysis

**Figure 1:**
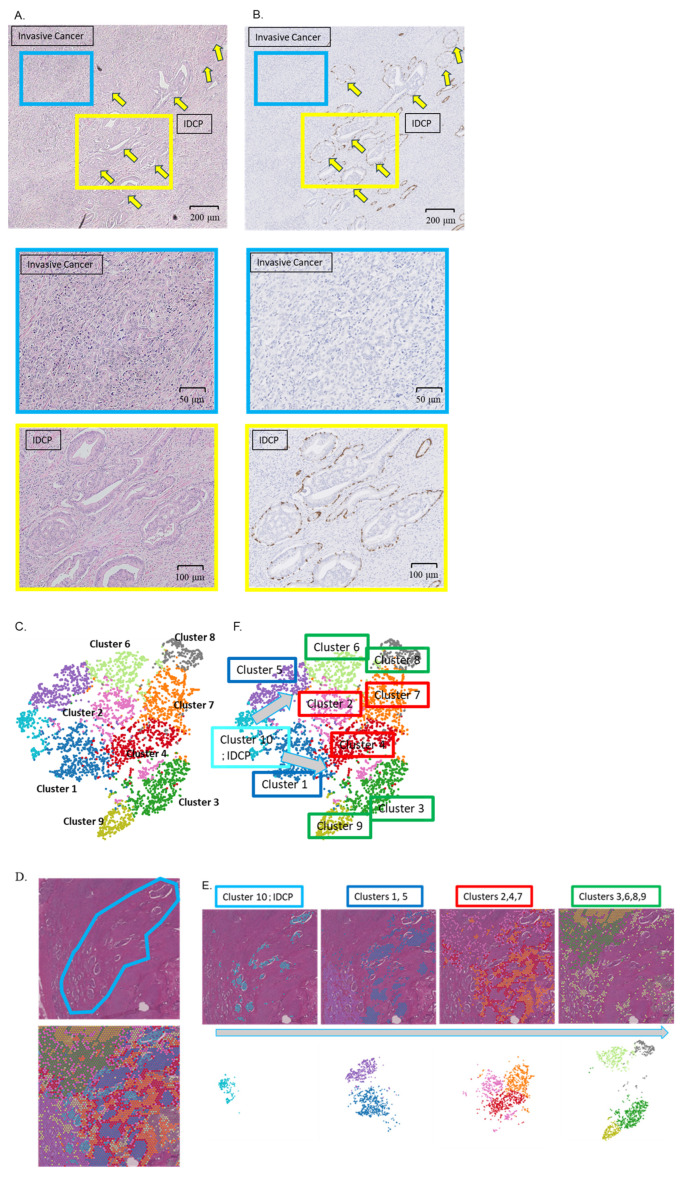

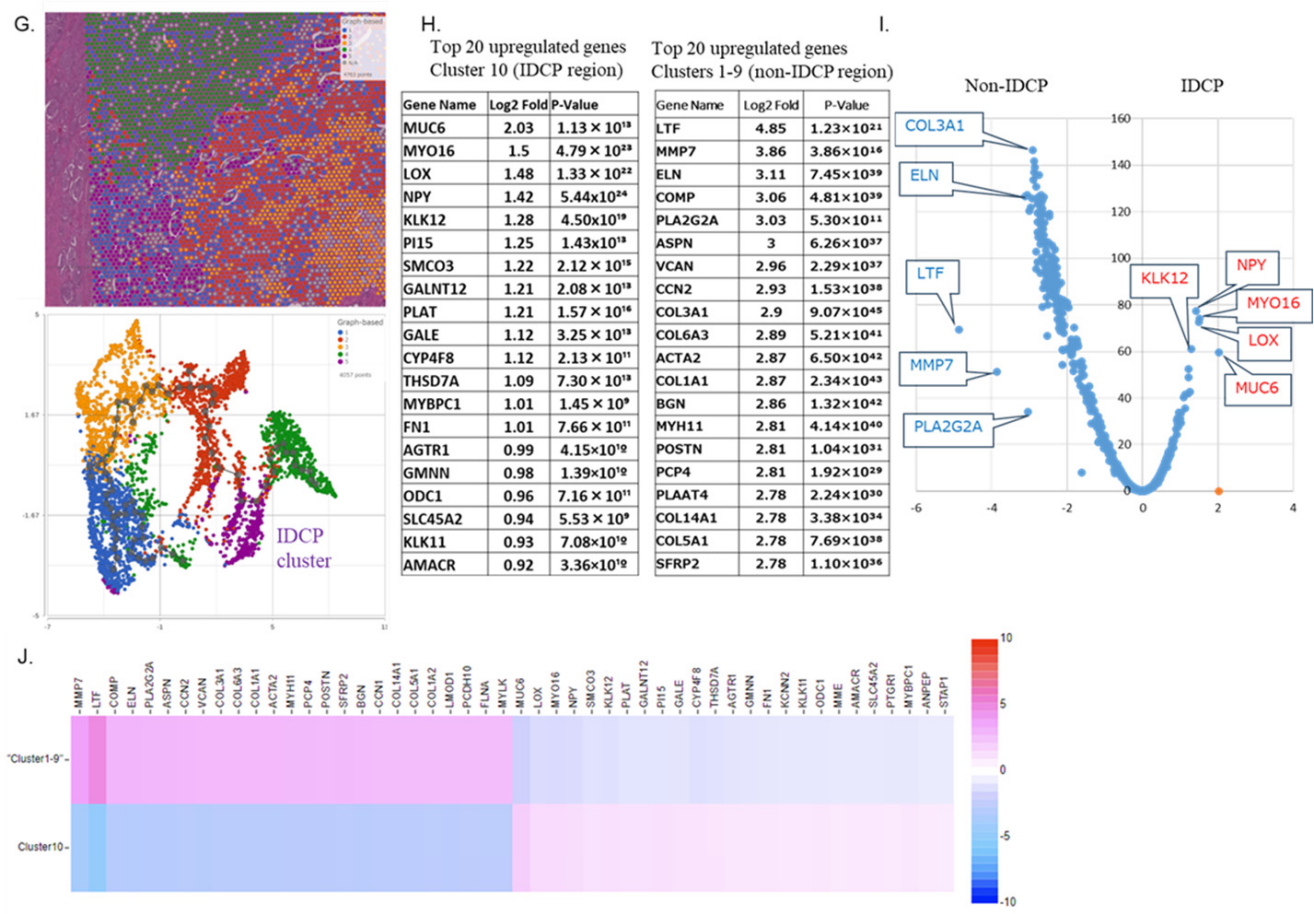
Diagnosis of IDCP and clustering through spatial gene expression analysis. (**A**) A 62-year-old man, diagnosed with prostate adenocarcinoma (cT3aN0M0, high risk, PSA 31.5 ng/mL), underwent a robot-assisted laparoscopic prostatectomy. HE staining revealed tumor invasion within normal glandular ducts with surrounding invasive carcinoma (blue square: invasive cancer area; yellow square: IDCP area; yellow arrow: IDCP). (**B**) Basal cell staining was performed on a formalin-fixed paraffin-embedded slide of the total prostatectomy specimen with a prostate cancer background showing Gleason Score 4 + 5. A cribriform morphological growth of the tumor was found in the normal glandular ducts with preserved basal cells, and IDCP was diagnosed (blue square: invasive cancer area; yellow square: IDCP area; yellow arrow: IDCP). (**C**,**D**) Spatial gene expression analysis (CytAssist Visium) classified the cells of the prostate tissue into 10 clusters. Of these 10 clusters, cluster 10 matched the IDCP region. (**E**,**F**) The clusters of invasive cancer lesions outside normal glandular ducts were close to those of IDCP on the pathology slides and were also close to IDCP clusters on the t-SNE plot, suggesting a similar gene expression pattern. (**G**) Trajectory analysis showed that IDCPs were similar in lineage to the neighboring invasive carcinomas. (**H**) The 20 most highly expressed genes in the IDCP (cluster 10) and non-IDCP regions (clusters 1–9). (**I**) The volcano plot shows the highly expressed genes in the IDCP and non-IDCP regions (**H**) Heat maps showed differences in gene expression between the IDCP and non-IDCP regions. (**J**) The heat map illustrates a clear distinction between gene expression in IDCP regions (cluster 10) and non-IDCP regions (clusters 1–9).

A 62-year-old man, diagnosed with prostate adenocarcinoma (cT3aN0M0, high risk, PSA 31.5 ng/mL), underwent a robot-assisted laparoscopic prostatectomy. Basal cell staining was performed on FFPE slides of total prostatectomy specimens with a Gleason score 4 + 5 prostate cancer background. A cribriform morphological growth of the tumor was evident in the normal glandular ducts with preserved basal cells, leading to a diagnosis of IDCP (Figure 1A,B). Spatial gene expression analysis (CytAssist Visium) categorized the prostate tissue cells into 10 clusters, with cluster 10 identified as an independent cluster corresponding to the IDCP region (Figure 1C,D). The clusters of invasive cancer lesions close to IDCP on the pathology slides exhibited gene expression patterns similar to those of the IDCP clusters on the t-SNE plot, whereas distant clusters showed distinct patterns (Figure 1E,F). A trajectory analysis further confirmed the lineage similarity between IDCPs and the neighboring invasive carcinomas (Figure 1G).

The 20 most highly expressed genes in the IDCP (cluster 10) and non-IDCP regions (Clusters 1–9) are shown in Figure 1F. Notably, *MUC6*, *MYO16*, *NPY*, and *KLK12* emerged as highly expressed genes defining the IDCP clusters, in contrast to genes from the tumor microenvironment, such as *LTF*, *MMP*, *ELN*, and *COL3A1*, which were relatively highly expressed in the non-IDCP clusters (Figure 1H). The volcano plot illustrates highly expressed genes in the IDCP and non-IDCP regions (Figure 1I). Additionally, heat maps underscored the differences in gene expression between the IDCP and non-IDCP regions (Figure 1J).

### 2.2. Visualization of Epithelial Cell, Androgen Receptor (AR) Signature Gene, and Other Upregulated Gene Marker Expression in the IDCP Region

**Figure 2:**
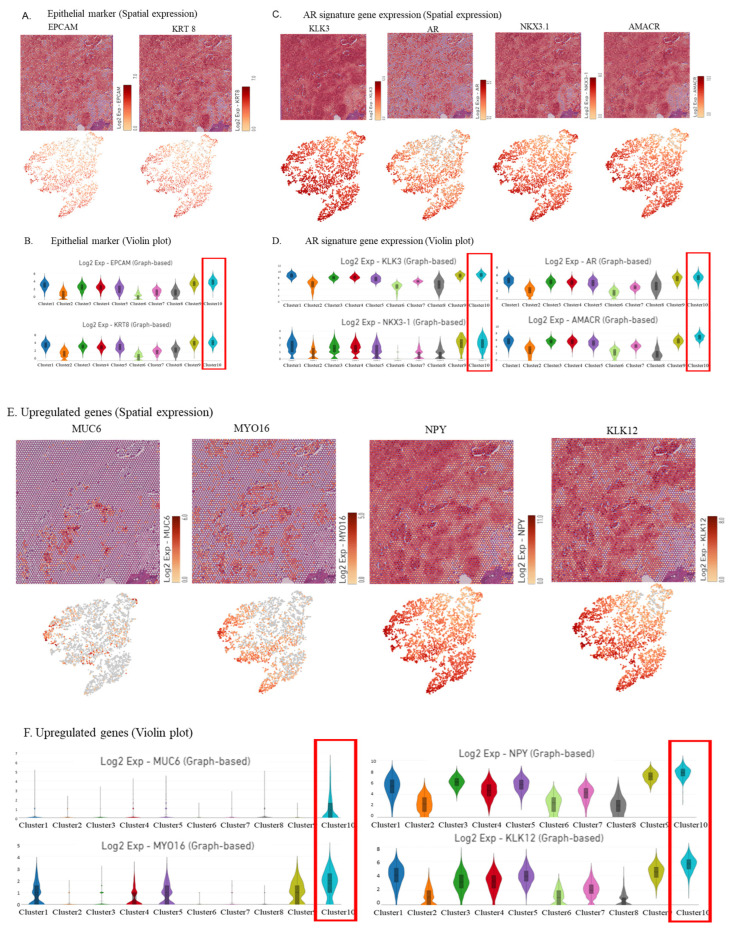
Visualization of the expression of epithelial marker genes, AR signature genes, and other upregulated genes in the IDCP region. (**A**,**B**) Spatial gene expression analysis showing that the epithelial markers were upregulated in all clusters (**A**), with similar findings in the violin plot (**B**). (**C**,**D**) Spatial gene expression analysis (**C**) and violin plot (**D**) showing the expression of a group of AR signature genes. (**E**,**F**) Spatial gene expression analysis (**E**) and violin plots (**F**) demonstrating the expression of MUC6, MYO16, NPY, and KLK12.

The spatial gene expression analysis revealed an upregulation of epithelial markers EPCAM and KRT8 across all clusters (Figure 2A), consistent with the findings from the violin plots (Figure 2B). Moreover, the examination of gene expression and violin plots demonstrated a high expression of a group of AR signature genes, including *KLK3*, *AR*, *TMPRSS2*, *NKX3.1*, and *AMACR* across all clusters (Figure 2C,D), indicating their tumor origin.

**Figure 3:**
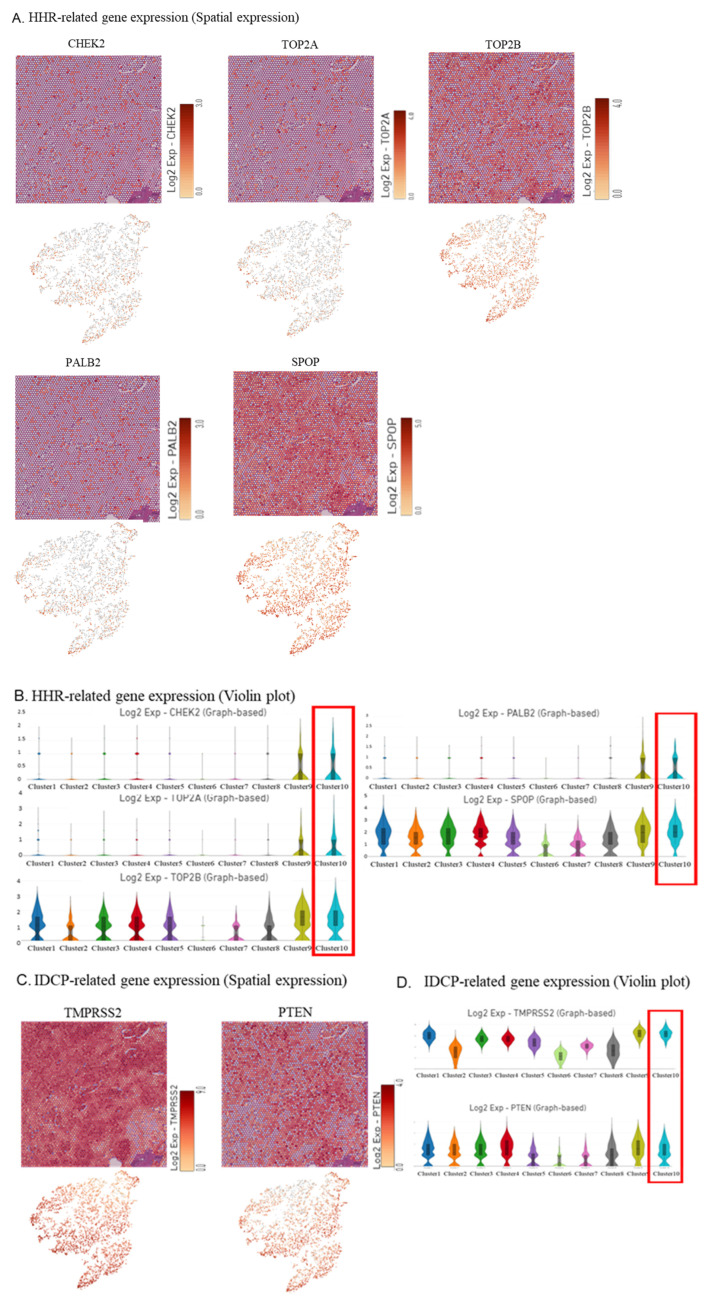
Visualization of the expression of HRR genes. (**A**,**B**) Spatial gene expression analysis (**A**) and violin plot (**B**) showing homologous recombination repair (HRR) gene expression. (**C**,**D**) Spatial gene expression analysis (**C**) and violin plot (**D**) showing TMPRSS2 and PTEN expression.

Furthermore, spatial gene expression analysis and violin plots showed the upregulation of *MUC6*, *MYO16*, *NPY*, and *KLK12* in the IDCP region, with expression diminishing as the distance from the IDCP region increased (Figure 2E,F). Additionally, spatial gene expression analysis and violin plots revealed a relatively high expression of homologous recombination repair (HRR) genes, such as *CHEK2*, *TOP2A*, *TOP2B*, *PALB2*, and *SPOP*, in the IDCP region (Figure 3A,B). However, the upregulation of *BRCA1* or *BRCA2* was not observed.

*TMPRSS2*, a known IDCP-associated gene, was highly expressed at the IDCP site without any evidence of *PTEN* downregulation (Figure 3C,D).

### 2.3. Visualization of the Expression of IDCP Fibroblast Markers, Immune Cell Markers, and Hypoxia Markers

**Figure 4:**
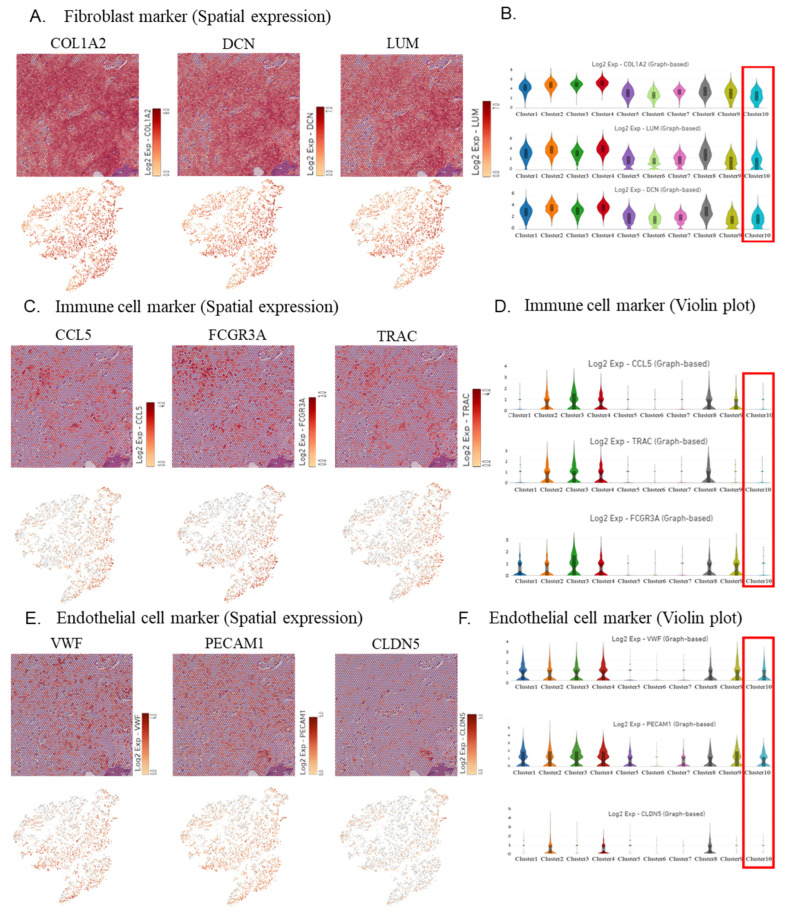

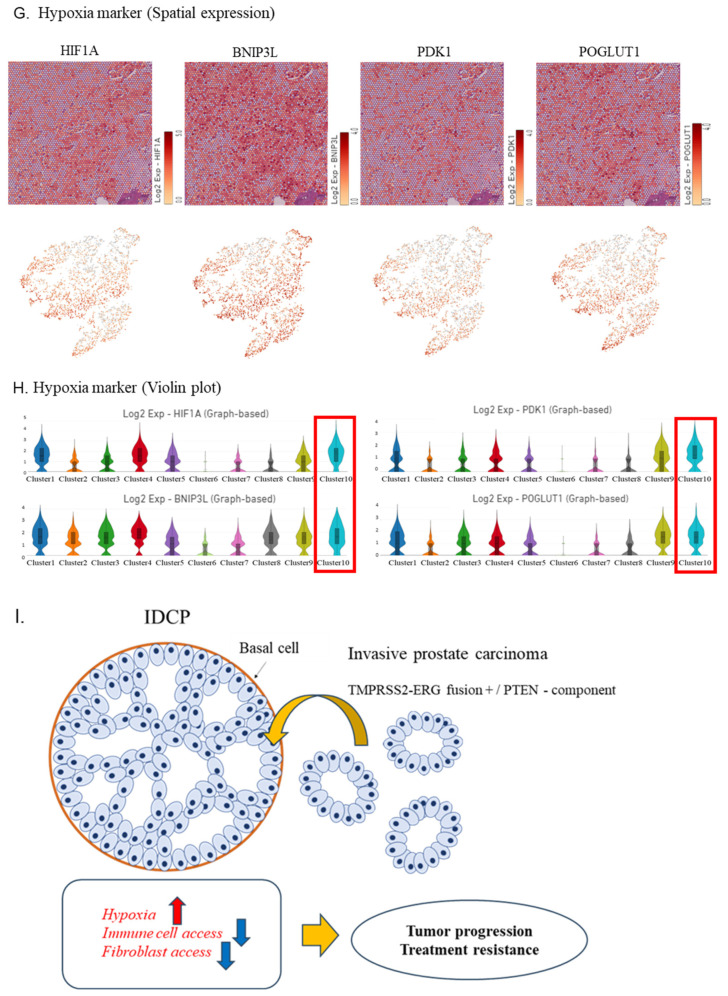
Visualization of the expression of fibroblast, immune cell, endothelial cell, and hypoxia markers. (**A**,**B**) Spatial gene expression analysis (**A**) and the violin plot (**B**) showing fibroblast marker gene expression. (**C**,**D**) Spatial gene expression analysis (**C**) and the violin plot (**D**) showing immune cell marker gene expression. (**E**,**F**) Spatial gene expression analysis (**E**) and the violin plot (**F**) showing endothelial cell marker gene expression. (**G**,**H**) Spatial gene expression analysis (**G**) and the violin plot (**H**) showing hypoxia marker gene expression. (**I**) Intraductal carcinoma of the prostate may enhance malignancy and increase resistance to therapy by causing hypoxia and a reduced recruitment of immune cells caused by its morphological features.

Spatial gene expression analysis and violin plots indicated the downregulation of the fibroblast markers *COL1A2*, *DCN*, and *LUM* in the IDCP cluster (Figure 4A,B). Moreover, spatial gene expression analysis and violin plots demonstrated a low expression of the immune cell markers *CCL5*, *FCG3A*, and *TRAC* in the IDCP cluster (Figure 4C,D). Spatial gene expression analysis and violin plots revealed no significant differences in the epithelial cell markers between the clusters (Figure 4E,F). Additionally, the analysis of the gene expression and violin plots revealed a high expression of the hypoxia markers *HIF1A*, *BNIP3L*, *PDK1*, and *POGLUT1* in the IDCP cluster (Figure 4G,H). This suggested that the loss of immune cells and the accumulation of hypoxic markers limited to the IDCP site may be attributed to the anatomic isolation of the IDCP site.

## 3. Discussion

IDCP is a significant pathological finding with clinical prognostic implications. Haffner et al. conducted a microdissection of both IDCP and surrounding invasive carcinoma sites and discerned site-specific gene expression patterns through their comparison. Consequently, they proposed a retrograde colony formation model wherein the *TMPRSS2-ERG* fusion gene-positive/*PTEN* loss component of invasive carcinoma infiltrates normal glandular ducts, forming IDCPs [2]. However, the precise sampling of lesion sites via microdissection is challenging and labor-intensive. Through single-cell analysis, Wong et al. compared cribriform carcinoma tissues with benign prostate tissues to elucidate the gene expression characteristics of cribriform prostate cancer [20]. Nonetheless, this study associated invasive cribriform carcinoma with IDCP but did not examine IDCP-specific gene abnormalities. Moreover, single-cell analyses lack spatial information, which impedes the identification of IDCP-derived cells. Therefore, we endeavored to identify IDCP-specific gene aberrations using spatial gene expression analysis.

An accurate diagnosis of IDCP is imperative for suitable treatment. IDCP has a significant prognostic value even in low-grade prostate cancer and should not be disregarded [21,22,23]. However, the classification of precursor-like (isolated) IDCP, a borderline lesion resembling HGPIN without a discernible cribriform pattern, remains contentious [24]. Therefore, in our study, we included samples from patients with a typical IDCP morphology, associated with high-grade prostate cancer. Following the IDCP diagnosis by two pathologists at our institution, the final diagnosis was confirmed by a pathologist in the United States who was experienced in diagnosing IDCP. The diagnosed case presented a typical IDCP morphology with surrounding invasive carcinoma, featuring a Gleason Score of 4 + 5 and a cribriform pattern tumor infiltrating the normal glandular ducts while retaining basal cells [25].

Spatial gene expression analysis revealed genetically similar tumors in and around the IDCP site. These tumors were *TMPRSS2*-positive but did not exhibit *PTEN* downregulation. As spatial gene expression analysis cannot identify mutations, potential *PTEN* mutations may have been overlooked. Nevertheless, genes upregulated at the IDCP site resembled those in clusters 1 and 5 adjacent to the IDCP. These findings support the hypothesis that the surrounding invasive carcinoma infiltrates normal glandular ducts. It is speculated that tumor cell clusters that have acquired high invasiveness form IDCP by invading and proliferating in normal glandular ducts with low tumor density.

*MUC6*, *MYO16*, *NPY*, and *KLK12* were upregulated at the IDCP site. *MUC6*, which encodes a member of the mucin protein family, is an organ-specific antigen and plays a pivotal role in epithelial surface cryoprotection. Some researchers insist that *MUC6* serves as a tumor marker in gastric and other cancers [26,27]. Compared to other *KLK* genes, both *KLK6* and *KLK12* are associated with increased invasive potential [28,29]. *MYO16*, which encodes the myosin XVI protein, regulates neuronal morphogenesis [30]. In addition, *NPY* (neuropeptide Y), a member of the *NPY* family, is widely expressed in the central nervous system, and its receptor, *NPY-1R*, is associated with the proliferative potential of prostate cancer [31,32]. These results indicate that IDCP may be involved in prostate cancer neuroendocrine differentiation, although the NE signature markers *CHGA*, *SYP*, *NCAM1 (CD56)*, *NKX2.1*, *MYCN*, and *AURKA* were not elevated in the present case.

Heterozygous deletions of *PTEN*, *TP53*, and *RB1* are important genetic alterations associated with neuroendocrine prostate cancer (NEPC), and are frequently observed in IDCP, suggesting possible molecular similarities between NEPC and IDCP. In a limited case series, Ikeda et al. observed the components of IDCP in nine patients with NEPC for whom tissue specimens were available at diagnosis [33]. Thus, the identification of IDCP may serve as a potential predictor of NEPC development; however, the association between IDCP and NEPC remains unclear and warrants further investigation [34].

IDCP upregulates several homologous recombination repair (HRR) genes. In the present case, an increased expression of *TOP2A*, *TOP2B*, and *SPOP* was observed. In a previous report, the accumulation of *TOP2A* induced by *SPOP* mutations was found to facilitate prostate cancer progression through the accumulation of DNA damage, and etoposide was found effective against SPOP-mutated prostate cancer [35].

In the present case, TOP2A upregulation and SPOP expression suggested the possible efficacy of TOP2A inhibitors. Chek2 and Palb2 were mildly upregulated at IDCP sites, but no overall upregulation was observed. Also, the high expression of HRR gene abnormalities suggested the potential efficacy of PARP inhibitors.

The decreased expression of fibroblast markers *COL1A2*, *DCN*, and *LUM*, and immune cell markers *CCR5* and *FCGR3A* at the IDCP site probably reflects a reduced recruitment of fibroblasts and immune cells owing to the absence of stroma caused by the anatomic isolation of IDCP. Fibroblast marker genes associated with stromal fibrosis contribute to enhance malignancy through the proliferation of cancer-associated fibroblasts (CAFs) in various cancers [36,37,38,39]. However, considering recent findings regarding the presence of cancer-promoting and cancer-suppressing fibroblasts [40], decreased numbers of tumor-suppressing fibroblasts and immune cells in the IDCP region may contribute to enhance malignancy. Furthermore, the elimination of immune cells from the inherently “immune-cold” tumor microenvironment of prostate cancer may render immune checkpoint inhibitors ineffective [41]. Furthermore, IDCP sites exhibited increased levels of hypoxia markers such as *HIF1A*, *BNIP3L*, *PDK1*, and *POGLUT1*. IDCPs growing within narrowly isolated normal glandular ducts are susceptible to hypoxia, which induces tumor cell starvation and hypoxic stress, increases glycolytic metabolism and angiogenesis, and increases the potential for metastasis and invasion. Thus, IDCP may enhance malignancy and resistance to therapy by inducing hypoxia and a reduced recruitment of fibroblasts and immune cells owing to the absence of stroma caused by its morphological features (Figure 4I). Focusing on the hypoxic state of IDCP, patients with prostate cancer and IDCP may benefit from inhibitors targeting *HIF1A*. In the present study, no increase in endothelial cell marker levels was observed despite the presence of hypoxia at the IDCP site. This implies that IDCP is an isolated and anomalous lesion, in which angiogenesis is less likely to be induced.

As this was a comparative analysis between IDCP and tumor sites with similar gene expression levels, identifying dramatic differences in gene expression was difficult. However, this study clearly demonstrated that IDCP can be recognized as a distinct cluster that tends to show the characteristic expression of markers related to immune cells, fibroblasts, and hypoxia. Because this was a single case study, the need to examine more cases in the future is acknowledged. As spatial gene expression analysis is dot-based, each dot contains the gene expression of approximately 10 cells. In fact, all 10 clusters of tumor cells classified in this study contained a mixture of immune cell and fibroblast markers. Integrating single-cell analysis data using the same sample through bioinformatics and introducing high-resolution spatial gene expression analysis at the single-cell level is thus necessary to perform a more accurate analysis. Furthermore, CytAssist Visium is not pure RNA-seq, but a technique that uses prepared probes to detect gene expression. Therefore, it cannot detect gene alterations such as TMPRSS-ERG2 fusion genes or PTEN mutations. This is another drawback of the CytAssist Visium and why it is necessary to integrate RNA-seq, including single-cell RNA-seq.

## 4. Materials and Methods

### 4.1. IDCP Diagnosis

We retrospectively reviewed patients who underwent total prostatectomy for PCa at Ehime University Hospital and selected suspected IDCP cases. For these patients, immunohistochemistry was performed on 10% neutral buffered formalin-fixed and paraffin-embedded surgical tissue samples, which were sectioned on a microtome (3–5 μm thick) and stained according to standard protocols. An anti-basal cell antibody (p63 antibody + HMW cytokeratin antibody) purchased from Proteintech (Rosemont, IL, USA) was used to stain the basal cells, and Hoechst 33,342 (dilution 1:2000; Molecular Probes, Eugene, OR, USA) was used to stain the nuclei.

Three pathologists from Ehime University Hospital, including a skilled US pathologist specializing in IDCP diagnosis, reviewed the pathology reports to identify cases suggestive of IDCP and to determine the presence of IDCP indicators. Finally, one case was used for the study.

This study was approved by the Institutional Review Board of Ehime University (Nos. 2108006, 2109014, and 2205001) and was conducted according to the principles of the Declaration of Helsinki. Written informed consent was obtained from the patient for the publication of the case report and accompanying images.

### 4.2. Spatial Transcriptomics (CytAssist Visium)

Spatial transcriptomics analyses were performed as previously described (Watanabe et al. 2023) [42]. Formalin-fixed, paraffin-embedded (FFPE) samples that passed the RNA quality control (DV200 > 50%) were used for spatial transcriptomic construction and sequencing. The tissues were prepared according to the Visium CytAssist Spatial Gene Expression for FFPE-Tissue Preparation Guide (CG000518, 10× Genomics, Pleasanton, CA, USA). The resected prostate tissue specimen was thinly sliced to 10 µm and CytAssist Visium was performed on a designated area of 6.5 mm × 6.5 mm square, including the IDCP site and the invasive cancer site. The tissue was permeabilized and the mRNA was reverse-transcribed into cDNA with barcode containing slide location information. cDNA was then amplified and sequenced to generate spatially resolved gene expression data. One type of barcode was assigned to each spot to enable mRNA analysis while preserving positional information. Sequencing was performed at the Research Institute for Microbial Diseases, Osaka University. Libraries were sequenced using an MGI DNBSEQ-G400RS (MGI Tech Co., Shenzhen, China). The Space Ranger pipeline v2022.0705.1 (10× Genomics, Pleasanton, CA, USA) and the GRCh38-2020-A reference were used to process FASTQ files. The sequencing results were guaranteed to be accurate as follows.

Number of reads: 366,120,606; number of spots under tissue: 4763; median genes per spot: 5665; mean number of reads under tissue per spot: 70,197.

t-SNE plots and violin plots were run and plotted using the Loupe Browser (10× genomics, Pleasanton, CA, USA). Trajectory and pathway enrichment analyses were performed and plotted using Partek flow software version 11 (Partek Incorporated, Chesterfield, MO, USA).

## 5. Conclusions

Spatial gene expression analysis could identify IDCP-specific gene expression profiles effectively in a typical IDCP case. Our findings suggest that a reduced recruitment of fibroblasts and immune cells and the absence of stroma due to the morphologic features of IDCP may contribute to enhance malignancy and treatment resistance. Overall, this study will help elucidate valuable markers and malignant mechanisms for diagnosing prostate cancer cases with IDCP and provide novel personalized treatment options. In the future, we aim to further elucidate IDCP-specific diagnostic markers and their developmental mechanisms by increasing sample size, integrating single-cell analysis data, and introducing high-resolution spatial gene expression analysis techniques.

## Data Availability

The data supporting the findings of this study are available from the corresponding author upon reasonable request. The raw and processed data of spatial transcriptomics generated in this study are openly available in GEO at https://www.ncbi.nlm.nih.gov/geo/, GEO number {GSE262276}.
